# CONTEXT MATTERS: THE IMPACT OF ENVIRONMENT, MOOD, AND FATIGUE ON MOBILE COGNITIVE ASSESSMENTS

**DOI:** 10.1093/geroni/igae098.2214

**Published:** 2024-12-31

**Authors:** Hannah Wilks, Matthew Welhaf, Andrew Aschenbrenner, John Morris, Jason Hassenstab

**Affiliations:** Washington University in St. Louis, St. Louis, Missouri, United States; Washington University in St. Louis, St. Louis, Missouri, United States; Washington University School of Medicine, St. Louis, Missouri, United States; Washington University in St. Louis, St. Louis, Missouri, United States; Washington University in St. Louis, St. Louis, Missouri, United States

## Abstract

To better understand the aging process and the subtle cognitive changes that occur in preclinical Alzheimer disease (AD), researchers are using technology to remotely measure cognition. We examined how concurrent environmental context, mood, and fatigue are associated with cognition in older adults at risk for AD. Participants (N = 288) were recruited from the Knight Alzheimer Disease Research Center (Mage = 75, RangeAge= 61-97). Cognition (e.g., associate memory and processing speed), context (e.g., with whom, where, activities prior to assessment), and current affect (e.g., mood, and fatigue) were measured contemporaneously on a smartphone-based application up to 28 times over a week in naturalistic environments. Clinical status was determined using the Clinical Dementia Rating □ (CDR□). Cognition at each assessment was predicted by contextual- and affect-related factors, and their interaction with CDR, while controlling for age, gender, education, and time in study. Associate memory and processing speed were worse in the presence of others, when assessed in locations other than home, and after engaging in sedentary behavior. Affective states were not associated with cognition. These effects were magnified in participants with very mild dementia symptoms, particularly for processing speed. Environmental and affective factors can influence memory and processing speed in older adults at risk for AD. Distracting environments were particularly problematic for individuals currently experiencing mild dementia symptoms. Studies of AD risk that rely on remote, unsupervised cognitive assessments should carefully assess and consider concurrent contextual factors when examining cognition.

